# Functional Technical Textile-Based Polymer Nanocomposites with Adsorbent Properties of Toxins and Dyes also Have Antibacterial Behavior

**DOI:** 10.3390/ma17123007

**Published:** 2024-06-19

**Authors:** Marlene Andrade-Guel, Christian J. Cabello-Alvarado, Carlos Alberto Ávila Orta, Gregorio Cadenas-Pliego, Brenda Cruz-Ortiz

**Affiliations:** 1 Centro de Investigación en Química Aplicada, Saltillo 25294, Coahuila, Mexico; carlos.avila@ciqa.edu.mx (C.A.Á.O.); gregorio.cadenas@ciqa.edu.mx (G.C.-P.); 2 México CONAHCYT-CIQA, Av. Insurgentes Sur 1562, Col. Credito Constructor, Alcaldía Benito Juárez, CDMX 03940, Mexico; 3 Facultad de Ciencias Químicas, Universidad Autónoma de Coahuila, Saltillo 25280, Coahuila, Mexico; b.cruz@uadec.edu.mx

**Keywords:** Nylon 6, non-woven fabric, adsorption, toxins, dyes, *E. coli*

## Abstract

This is the first study of non-woven fabrics elaborated by melt-blowing from polymer nanocomposites made of Nylon 6 and nanoclay (Cloisite 20A) modified with an amine (1,4 diaminobutane dihydrochloride). Morphological and physical characteristics, adsorption capacity, and antibacterial properties are presented. From the X-ray diffraction (XRD) results, it was possible to observe a displacement of the signals to other 2θ angles, due to an α to ϒ phase shift. The scanning electron microscopy (SEM) images showed that the mean diameter of fiber decreased as the content of nanoclay increased. The mechanical tests showed that the tear strength force of neat nylon was 1.734 N, but this characteristic increased to 2.135 N for the sample with 0.5% modified nanoclay. The inulin adsorption efficiency of the Nylon 6/C20A 1.5% and Nylon 6/C20A 2% samples at 15 min was 75 and 74%, respectively. The adsorption capacity of Nylon 6/C20A 1.5% and Nylon 6/C20A 2% for methylene blue and methyl orange remained above 90% even after four adsorption cycles. In addition, non-woven fabrics present antibacterial activity against *E. coli*.

## 1. Introduction

Functional textiles are adapted to address the different necessities of human beings. Functional textiles showed a growth rate of 30% from 2015 to 2020 due to support and investment from different industries, which seek new technological developments in this area [1]. Textiles made from fibers with micrometric or nanometric sizes are attractive in different application areas. Three critical areas are energy generation, medicine, and environmental care [2].

On the other hand, adsorbent nanomaterials can have different characteristics and be incorporated into a polymer matrix to make composite materials and apply them in technical textiles.

Some adsorption studies with carbon-based materials from potato waste have had good results in the adsorption of dyes such as allura red, carmine, and tartrazine; these materials present different behaviors of monolayer and multilayer adsorption mechanisms [3]. It has also been seen that other materials designed and manufactured with nanometric dimensions (MXene nano sheets) effectively eliminate cationic and anionic azo dyes (methylene blue and Congo red) through adsorption processes [4,5]. These nanometric adsorbent materials can be incorporated into a polymeric matrix and form fibers for application as an adsorbent at an industrial level.

Moreover, functional textiles applied in medicine are promising products that help drug administration; specifically, they can improve the dermal penetration capacity of the active molecule and avoid the risk of toxicity. Another application is in housing medical devices for hemodialysis [6,7]. Koh et al. reported the fabrication of nanofibers for hemodialysis consisting of three layers of nanofibers: the top layer of poly(methyl methacrylate)-graft-poly(dimethylsiloxane) (PMMA-g-PDMS) nanofiber, the second layer of polyamide 6 (PA6), and the third one containing nanofibers of polyamide/zeolite for creatinine removal [8]. In the hemodialysis process, it is necessary to remove some nitrogenous molecules in the blood; one is urea because it causes kidney problems and affects the liver [9]. Abidin et al. prepared hollow fiber membranes to combine filtration and an adsorption process, removing 39.2% of urea with a double-layer fiber of polysulfone and polymethyl methacrylate (N-PMMA) [10]. 

Recent studies on non-woven fabrics incorporating nanoparticles like graphene nanoplatelets and carbon black have achieved 90% and 80% urea adsorption, respectively [11,12]. Some functional textiles have been used as filtration systems for separation and cleaning in the environmental care area; their production has increased due to the increasing environmental demands to remove contaminants from either air or water [13,14]. Various types of contaminants (organic and inorganic) are released into the water. Among these contaminants, dyes stand out because they are toxic. Dyes such as methylene blue and methyl orange are persistent pollutants in aquifers because conventional water treatment methods cannot remove them. Therefore, new methods for removing these contaminants are needed [15,16]. 

Some textile studies have reported the removal of dyes of different natures. Dao et al. studied non-woven polyester fabrics as a support substrate with rGO (reduced graphene oxide) and Cu2 particles that form a photocatalytic membrane for the degradation of methylene blue, under the influence of natural sunlight. The manufacturing process of this compound was carried out in four stages, achieving the elimination of 96% of methylene blue in 120 min [17]. Rana et al. studied the incorporation of glycidyl methacrylate into polyethylene non-woven fabric, and carried out a chemical modification of it and applied it in the adsorption of methylene blue, achieving the elimination of 52% in the first 5 h [18].

Andrade et al. reported on non-woven fabric based on Nylon 6 and ZnO. To remove methylene blue and reduce antimicrobial performance, one of the experiments involved accelerated washing, which consists of observing the effects on antimicrobial properties [19].

It has been seen that another advantage of using nanoparticles in textiles as antibacterial agents is that it reduces the side effects of contact with the skin when compared to agents that are currently used commercially.

On the other hand, in recent years, our research group has used the ultrasonic-assisted extrusion method for incorporating nanoparticles into a non-woven fabric. In this methodology, a nanocomposite containing a polymer matrix and the nanoparticle as a reinforcement is first prepared. Then, the non-woven fabric using the melt-blowing method is manufactured. This technique helps the dispersion of the nanoparticles and does not allow the nanoparticles to detach with washing. Our research group studied the non-woven fabric of Nylon 6 with different nanoparticles such as ZnO, carbon black, and graphene.

The novelty is that this is the first study of non-woven fabrics elaborated by melt-blowing from polymer nanocomposites made of Nylon 6 and nanoclay (Cloisite 20A) modified with an amine (1,4 diaminobutane dihydrochloride) to evaluate its effect on the physicochemical properties and organic compound adsorption. Finally, this study evaluates the amine’s antibacterial properties.

## 2. Materials and Methods

### 2.1. Reagents

Nylon 6 from DuPont Zytel^®^7301 NC010, organoclay Cloisite C20A with a particle size < 10 µm and lamellar spacing of 2.7 nm. 1,4-diamino butane dihydrochloride purity of 99% was purchased from Sigma Aldrich products (St. Louis, MO, USA). Methylene blue (MB) (99%), methyl orange (MO) (99%), and inulin (98%) were purchased from Sigma Aldrich, urea (95%) was purchased from Faga Lab (Sinaloa, Mexico), and distilled water with a pH of 7 was used as a solvent to obtain the solutions.

### 2.2. Chemical Modification of Cloisite 20A by Ultrasonic Tip

Cloisite 20A modification with 1,4-diamino butane dihydrochloride was made following methodologies already reported by Andrade et al. [20]. The chemical modification treatment was carried out by dispersing 1 g of C20A nanoclay in 20 mL of distilled water, with 1,4-diaminobutane dihydrochloride, in a 1:1 ratio, using a homemade ultrasonic generator, with an output power of 750 W, at an amplitude of 50% and a variable frequency of 15 to 50 KHz, catenoidal ultrasonic tip (Branson Ultrasonics Corp., Brookfield, CT, USA; D, 51.27 cm). For safety reasons, the experiments were carried out in a soundproof cage. Ultrasound treatment time was 120 min and was used at room temperature. At the end of the experiments, the C20A clay was filtered and dried at 80 °C for 24 h.

### 2.3. Nanocomposite Preparation

The nanocomposites were prepared according to the method previously reported [21]. Polymer nanocomposite preparation was carried out using the ultrasound-assisted melt extrusion process (US) to homogenize the mixture of the particles within the polymer. For the extrusion process, a lab-size twin-screw extruder from Thermo Fisher Scientific (model, Prism TSE-24MC) with a screw diameter of 24 mm and L/D ratio of 40:1 was assisted by a catenoidal ultrasonic tip (Branson Ultrasonics Corp., CT; D, 51.27 cm). The extruder was connected to a homemade ultrasonic generator (15 to 50 kHz, 100% of 750 W). The temperature profile was a flat one, at 215 °C in all areas of the extruder, with a screw speed of 115 rpm. Nylon 6/C20A nanocomposites were prepared at different concentrations (0.25% wt, 0.5% wt, 0.75% wt, 1.5% wt, and 2% wt). These experimental conditions help increase the homogeneous size of the pellet. As a post-extrusion system, a cooling bath was used at the outlet of the die and a pelletizer (Thermo Fisher Scientific, Waltham, MA, USA).

### 2.4. Preparation of Non-Woven Fabric Materials 

**Figure 1:**
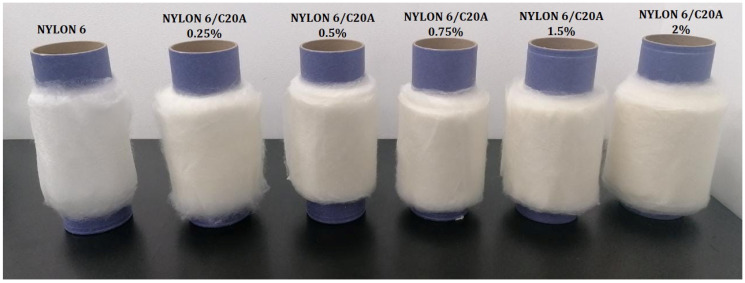
Identification of non-woven fabric samples.

Nylon 6/C20A non-woven fabrics were elaborated by fiber extrusion technology (FET-UK) pilot machine equipment. The profile temperatures were 240 °C extrusion zone 1; 245 °C extrusion zone 2; 245 °C extrusion zone 3; 250 °C extrusion zone 4; 255 °C flange zone; 255 °C melt pump zone; 255 °C dual heat zone; 255 °C melt blow adapter zone; and 255 °C melt blow hot air zone. Figure 1 shows the identification of samples, for example, (Nylon 6/C20A 0.25%) polymer = Nylon 6; C20A = Cloisite 20A modified with 1,4-diamino butane dihydrochloride; 0.25% = concentration of the modified nanoclays.

### 2.5. Characterization 

The morphology and elemental composition of the materials were characterized by scanning electron microscopy and energy-dispersive spectroscopy (SEM-EDS) through an electron microscope model JEOL model JSM-7401F (Jeol LTD., Akishima, Tokyo, Japan). The sample was coated with gold and palladium before analysis. 

XRD (X-ray diffraction) was performed by a Rigaku model Ultima IV Smartlab (Rigaku Beijing, China) diffractometer operating at 40 kV and 40 mA with stability of 0.01%/8 h with a scanning interval in the 2θ scale from 10 to 80°. 

The mechanical properties were measured following ASTM D2261 Standard norm (2013) standard test methods for tear strength properties of fabrics woven from stretch yarns. ASTM International standard tests were used to evaluate the tearing resistance of the non-woven fabric by the tongue (single rip) procedure. Tensile strength was carried out following ASTM D5034 Standard. These tests considered the machine direction (MD) and transversal direction (TD).

The equipment used for both the tear resistance and the resistance to breakage and elongation was a United Universal machine with an environmental chamber CEF-80, tensiometer model SFM-100KN from United Testing.

#### 2.5.1. Adsorption of Uremic Toxins

The preparation of urea and inulin solutions and their measurement and calculations followed the method described in previous works [12]. First, Nylon 6 and Nylon 6/C20A, 30 cm × 30 cm non-woven fabric specimens were placed in a prototype (see Figure 6c; the prototype is made of glass and is the actual size of a hemodialysis dialyzer) with the solutions of uremic toxins. Then, the solution was prepared, which consisted of a mixture of urea and inulin at a concentration of 390 mg/dL. The simulation of the hemodialysis process lasted 4 h, and every 15 min, an aliquot was taken to analyze in the UV-Vis spectrometer.

All experiments were performed in triplicate. The removal percentage of each toxin was calculated with the following equation.

(1) $$\%\;Removal=\frac{\left(Ci-Ce\right)}{Ci}\times 100$$

where *Ci* is the initial concentration and *Ce* is the final concentration. 

For the analysis of uremic toxin adsorption, the samples were analyzed in a UV-Vis spectrometer (Duetta Horiba Scientific, Beijing, China) at different wavelengths of 200 nm (urea) and 270 nm (inulin). The adsorption isotherms data were fitted and the correlation coefficient (R^2^) was calculated using the trendline command in Microsoft Excel 2016 for each isotherm. The Langmuir isotherm was calculated using the following equation.

(2) $$\frac{Ce}{q_{e}}=\frac{Ce}{q_{m}}+\frac{1}{K_{Lqm}}$$

where q_e_ (mg·g^−1^) and Ce (mg·L^−1^) are the concentrations of the solid and liquid phases of adsorbate in equilibrium, respectively, q_m_ is the maximum adsorption capacity, and K_L_ is the constant obtained from the graph of Ce/q_e_ against Ce.

The Freundlich isotherm was calculated using the following equation.

(3) $$\ln qe=\ln K_{F\;}+\left(\frac{1}{n}\right)\ln Ce$$

where K_F_ (mg·g^−1^) (L·mg^−1^) and 1/n are the Freundlich constants related to the adsorption capacity and *n* is the heterogeneity calculated by linearly plotting Inqe against InCe [22].

#### 2.5.2. Adsorption of Dyes (Methylene Blue and Methyl Orange)

Batch experiments followed the methodology reported previously [19]. The adsorption experiments were carried out by filtering 20 mL of the 200 mg/L solution (MO or MB) with non-woven fabric (circle 11.5 cm diameter) with a weight of 20 mg at room temperature for 60 min. The non-woven fabric was fixed in a glass funnel and the MB or MO solution was poured into the non-woven fabric slowly for 60 min. Every 10 min, an aliquot was taken and read in the UV-Vis spectrometer.

The residual concentration of the dye solution was determined using a calibration curve prepared at the corresponding maximum wavelength (465 nm MO) (590 nm MB) using a UV-Vis spectrometer (Duetta Horiba Scientific, Horiba, Beijing, China).

Adsorption capacity (mg/g) and removal efficiencies in the percentage of MB and MO dyes were calculated using Equations (1) and (2).

(4) $$q_{e}=\frac{\left(Ci-Ce\right)V}{m}$$

(5) $$\%adsorptionefficiency=\frac{\left(Ci-Ce\right)}{Ce}\times 100$$

where Ci and Ce are the initial and equilibrium MO and MB dye (mg) concentrations. V and m are the volume of dye solution and amount of adsorbent (g). Langmuir and Freundlich isotherms were plotted using these data.

#### 2.5.3. Reusability Study

Adsorbent materials must remain stable even after several adsorption cycles. The materials for the organic compounds adsorption were evaluated in four cycles, as described in Section 2.5.2, without prior material washing. The fabrics were dried at room temperature to conduct the following adsorption cycle to determine the material utilization.

#### 2.5.4. Antibacterial Activity

The antibacterial activity was evaluated against Gram-negative bacteria (*Escherichia coli*). The Kirby–Bauer method was used. The bacteria were incubated at 37 °C for 18 h, with an *E. coli* concentration equal to 0.5 McFarland Standard. Grown cultures were prepared and smeared onto the surface of nutrient agar and soy trypticase in Petri plates. Specimens of approximately 3 mm × 3 mm in size were cut from the textile samples under aseptic conditions and placed on the agar surface and the plates were incubated at 37 °C for 18 h. The standard antibiotic was gentamicin, the negative control was DMSO, and the diameter of the formed zone of inhibition (in mm) was determined.

## 3. Results and Discussion

### 3.1. X-ray Diffraction (XRD)

**Figure 2:**
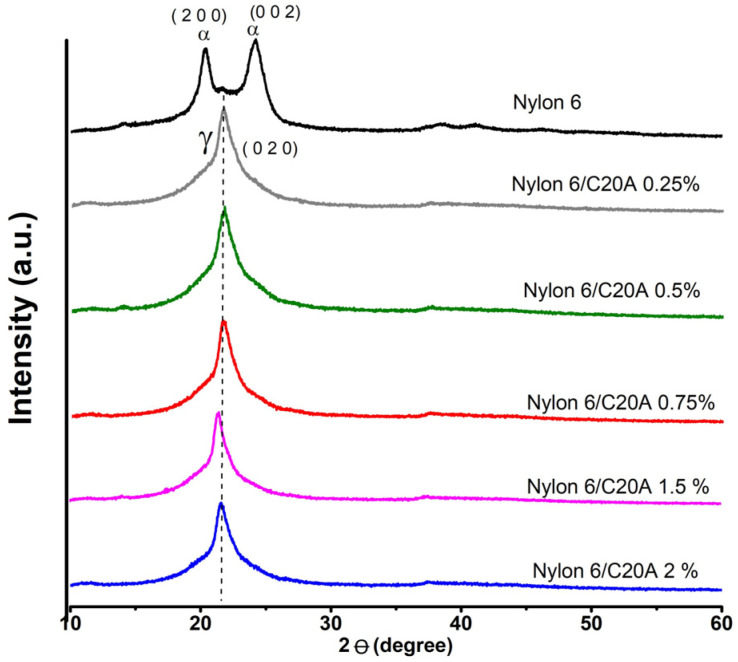
X-ray diffraction patterns of Nylon 6 non-woven fabric and Nylon 6/C20A non-woven fabric at different concentrations of nanoclay C20A (0.25, 0.5, 0.75, 1.5, and 2.0%).

Figure 2 shows Nylon 6 and Nylon 6 nanoclay C20A diffraction patterns with different clay contents (0.25, 0.5, 0.75, 1.0, and 2.0%). Neat Nylon 6 shows two strong signals at 20.33° and 24.19° corresponding to the α crystalline phase in the planes (2,0,0) and doublet in (2,0,2)/(0,0,2), respectively [23,24]. All Nylon 6 non-woven fabrics with C20A nanoclay at different concentrations show a pseudo-hexagonal ϒ crystalline phase, consisting of parallel chains with hydrogen bonds causing torsion on the molecular chains in zigzag planes [25]. Therefore, it seems that the incorporation of modified nanoclay changes the crystalline structure of the polymer matrix of the non-woven fabrics, probably because nanoclay acts as a nucleating agent and increases the crystallization rate. This change is observed even at the lowest content of nanoclay (0.25%). Another intervening process is the fusion heat used for the manufacture of the non-woven fabric since some reports referred that a polymer transformation with an increase in temperature and then cooling such as that of film formation causes a change in the crystallinity of Nylon 6 [26]. The diffraction peak centered at 21.77° (ϒ) in the non-woven fabric Nylon 6/C20A 1.5% shifts towards 21.23° as the nanoclay content increases. It is mainly due to the degree of intercalation of the nanoclay galleries in the polymer matrix, in addition to the dispersion that helps create a homogeneous distribution of the nanoclays in the nylon polymer matrix [20]. This peak for the non-woven fabric Nylon 6/C20A 2% appears at 21.5°. This observation agrees with Cabello et al., where the same shift in the crystalline phase was observed when the zeolite was incorporated [27].

### 3.2. Scanning Electron Microscopy (SEM)

**Figure 3:**
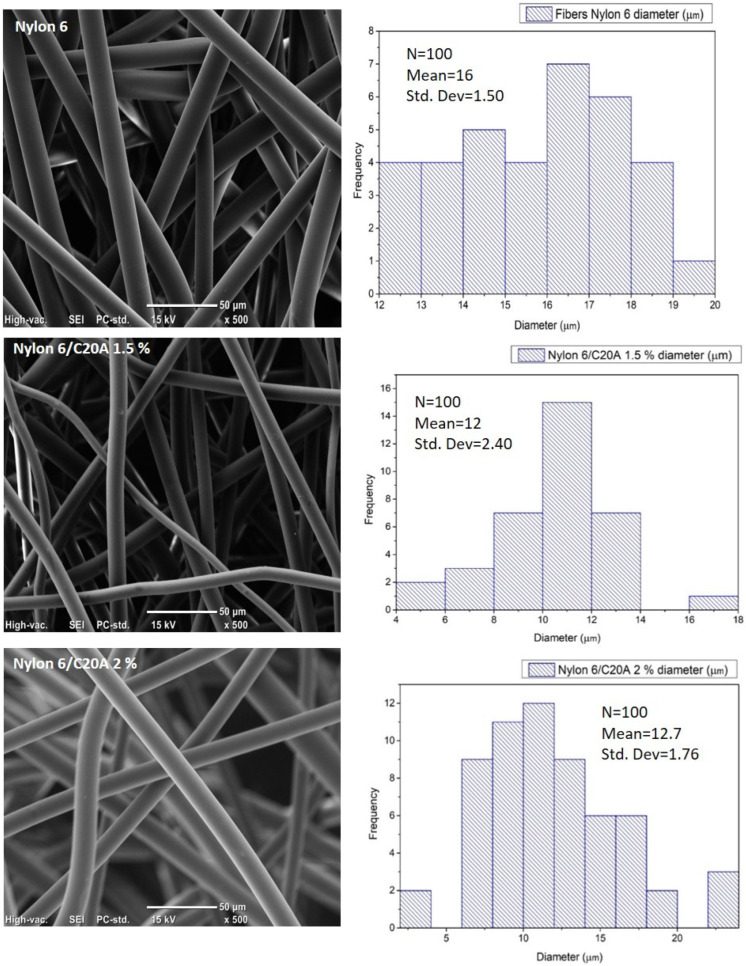
Frequency distribution and 500X SEM images of non-woven fabric of neat Nylon 6, Nylon 6/C20A 1.5%, and Nylon 6/C20A 2.0%.

The SEM-EDS analysis showed the morphological changes and elemental composition in the non-woven fabrics, for example, samples of neat Nylon 6 and Nylon 6 C20A (1.5 and 2.0%) (Figure 3). All samples are generally composed of uniform and smooth fibers randomly oriented, presenting a cylindrical shape (see Figure S1).

In addition, the fiber diameters distribution for the samples are as follows (Table 1). Figure 3 show frequency distribution for neat Nylon 6 fabric has a mean fiber diameter of 16 and 17 ± 1.5 µm. Nylon 6/C20A 1.5% has a mean fiber diameter of 12 ± 2.4 µm, and Nylon 6/C20A 2.0% has a main fiber diameter of 12.7 ± 1.76 µm. A decrease in the size of the fiber diameter is observed as the concentration of the nanoclay increases.

A reduction in the fiber diameter can be observed as the modified nanoclay content increases because the nanoclays have amino groups (modifying groups) that can increase the polymer fluidity acting as a lubricant, reducing the fiber size when it is collected. These results agree with the results reported by Fukushima et al. These authors reported an increase in the fluidity of composites of PE-TiO_2_ obtained by ultrasound-assisted extrusion attributed to the large exposed surface area of the particles which increased the mobility of the polymer chains [28].

**Figure 4:**
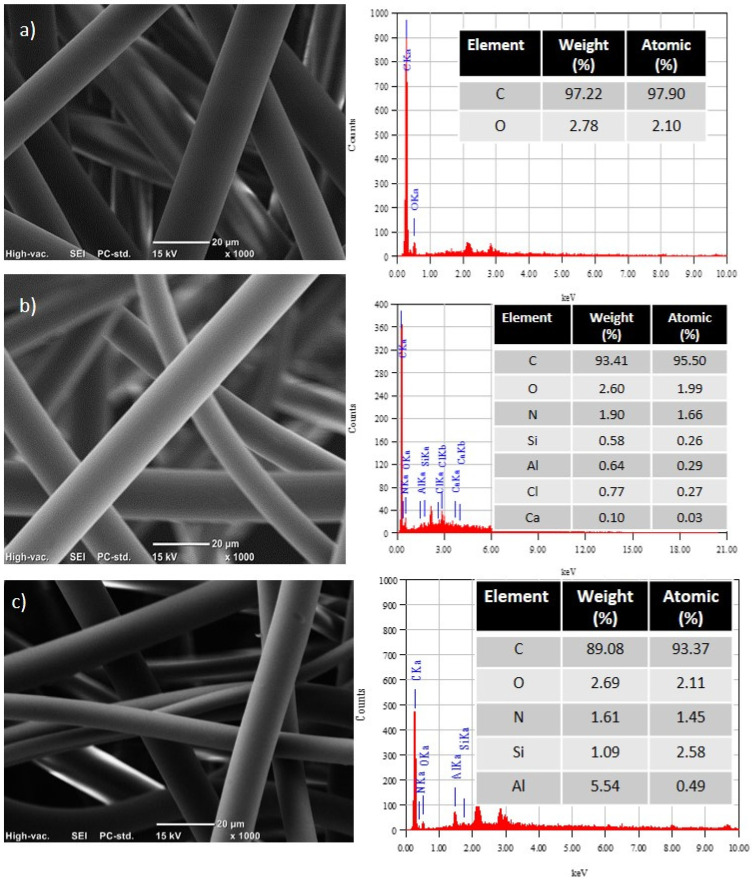
EDS and 1000X SEM images of non-woven fabric of (**a**) neat Nylon 6, (**b**) Nylon 6/C20A 1.5%, and (**c**) Nylon 6/C20A 2.00%.

EDS analysis was performed at 1000× magnification for neat Nylon 6 and Nylon 6/C20A 2.0% fabrics (Figure 4a,b). Neat Nylon only showed C and O elements. Nylon6/C20A 2.0% showed C at 0.27 KeV, N at 0.39 KeV, O at 0.52 KeV, Al at 1.48 KeV, Si at 1.73 KeV, Cl at 2.26 KeV, and Ca at 3.69 KeV. These elements are present in modified nanoclay and the polymer, as reported previously by Andrade-Guel et al. in PLA/C20A nanoclay nanocomposites [20].

### 3.3. Mechanical Tests

**Figure 5:**
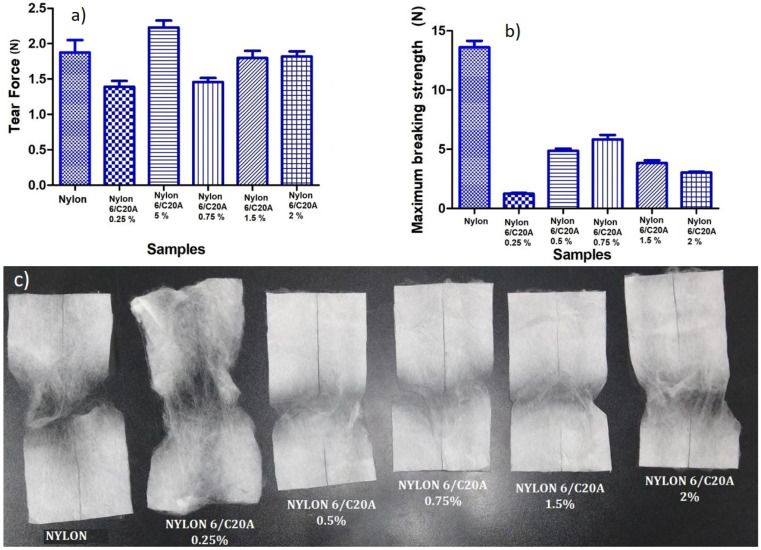
Mechanical properties of non-woven fabrics (**a**) maximum breaking strength, (**b**) tear force, and (**c**) images of fabrics after maximum breaking strength test.

The mechanical properties are shown in Figure 5 and Table 2. Neat Nylon 6 non-woven fabric has a tear force of 1.734 N; when the clay is added, the tear force decreases. 

The Nylon 6/C20A 0.5% shows a higher tear force than neat Nylon. This behavior can be related to an effective dispersion at low concentrations. Nanoclay can inhibit the mobility of the polymer chains, causing a decrease in rigidity [12]. Also, previous studies have reported that the interactions between the layers of chemically modified silicates and the polymeric matrix can drive improvements in mechanical properties.

Figure 5b shows the breaking strength of the non-woven fabrics, while Figure 5c shows the corresponding images after maximum breaking strength. In Table 2, it can be seen that the maximum breaking strength for Nylon 6 was 13.24 N, and the Nylon 6/C20A 0.25% sample reached its lowest point (1.19 N) when the modified nanoclay load increased to 0.75% wt, the maximum breaking strength increased to 5.56 N. This increase may be because at low concentrations, the nanoparticles act as breaking points and the polymer chains and cracks in the polymer flow faster, and by increasing the concentration of nanoclay, the polymer becomes more rigid and the cracks move with more difficulty at the crack sites. It has been shown that nanoparticles coated in a polymer matrix can prevent the formation of cracks or self-healing [29].

Regarding the morphological properties, they indicate that by increasing the concentration of modified nanoclay, the fiber diameter decreases; on the other hand, the mechanical properties benefited at concentrations of 0.5% and 0.75% of nanoclay, which indicates that there is a good performance when adding these additive contents.

### 3.4. Toxin Adsorption of Non-Woven Nylon 6/C20A

**Figure 6:**
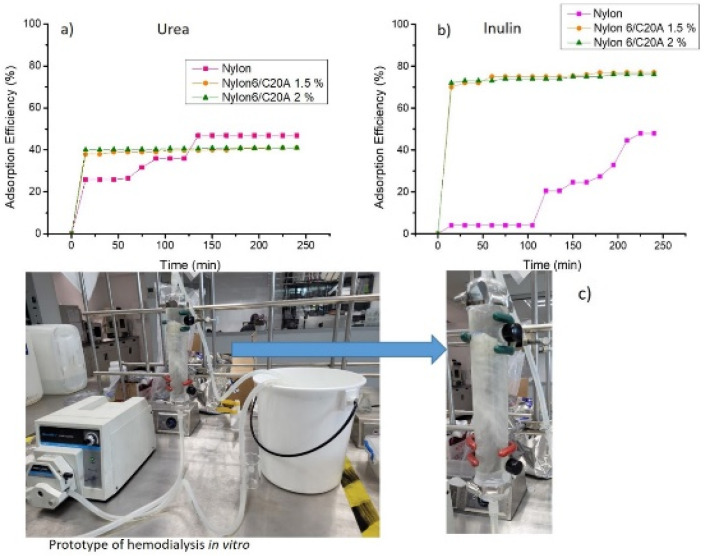
Adsorption profile over time of (**a**) urea and (**b**) inulin onto Nylon 6/C20A non-woven fabric (T = 37 °C, adsorbent dose= 380 mg/L), and (**c**) image of the hemodialysis in vitro prototype.

Figure 6 shows the adsorption of urea and inulin concerning contact time. The system equilibrium is achieved at the maximum adsorption efficiency. For urea (Figure 6a), an adsorption efficiency of 40% is achieved for the non-woven fabric of Nylon 6/C20A 1.5% and Nylon 6/C20A 2%. However, neat Nylon has an efficiency slightly above 47% because it has more significant active sites for the union with urea.

In a previous study, PLA-Cloisite 20A 5% presented an efficiency of 65%. This difference is due to the modified nanoclay amount. In the present study, we focus on evaluating non-woven fabric with a maximum nanoclay concentration of 2% [20]. Figure 6b shows the inulin adsorption: after 210 min, the maximum adsorption capacity of 48% is reached for neat Nylon 6. This result is in agreement with Andrade et al. [11]. Non-woven fabric Nylon 6/C20A 1.5% and Nylon 6/C20A 2% present similar behavior in the inulin adsorption process. After 15 min, 75 and 74% adsorption efficiencies are reached, respectively. These values indicate rapid adsorption because adsorption sites are generated by the modification of the nanoclay in the non-woven fabric surface. Figure 6c shows an image of the in vitro hemodialysis prototype. It is a continuous system where the non-woven fabric is placed into the device, and the adsorption efficiency is monitored for 4 h. 

The experimental adsorption data of urea and inulin in Nylon 6/C20A nanocomposites were fitted with the Langmuir and Freundlich models; the results are presented in Table 3 and Table 4, respectively. In Table 3, the correlation coefficient R^2^ of the Freundlich model is more significant than that of the Langmuir model, indicating that the experimental data coincide with the Freundlich model in which the adsorption of urea onto Nylon and all samples of Nylon 6/C20A at different concentrations is carried out in a multilayer process [22]. All samples have heterogeneous behavior because the adsorption process takes place in different layers, the mass transport of adsorbate from the solution to the inner surface of the porous adsorbent where adsorption occurs. The structure of the amine-modified nanoclay allows electrostatic interactions with urea, carrying out a chemical adsorption, not only a physical one.

By contrast, the adsorption of inulin (Table 4) adjusts better to the Langmuir adsorption model, indicating a homogeneous surface, where the occupied sites form a monolayer [30]. All samples have homogeneous behavior because the adsorption process takes place in a single layer. The inulin structure presents hydroxyl groups that interact with the amino acid groups of the modified nanoclay, and a monolayer process occurs due to the fact that the adsorption sites are occupied quickly. The adsorption process in an aqueous solution can occur through the porosity of the interconnected fibers in the non-woven fabric or chemically between the functional groups of the inulin and the adsorbent.

### 3.5. Dye Adsorption

**Figure 7:**
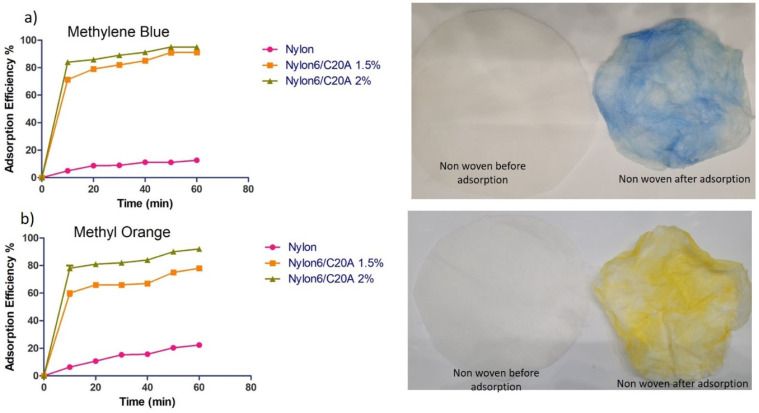
Time effect on the adsorption of (**a**) MB and (**b**) MO dye onto Nylon 6/C20A non-woven fabric (T = 25 °C, adsorbent dose = 200 mg/L); images of Nylon 6/C20A 2%.

Figure 7a shows the adsorption of methylene blue dye with respect to time. In the case of neat Nylon, after 20 min, the adsorption efficiency reaches 13%. Nylon 6/C20A nanocomposites show rapid adsorption in the first 10 min. Then, the adsorption capacity increases, reaching the equilibrium after 60 min, removing 95 and 91% for Nylon 6/C20A 1.5% and Nylon 6/C20A 2%, respectively. In addition, the figure shows images from before and after methylene blue adsorption in the fabrics. Chen et al. studied the methylene blue adsorption on polypyrrole nanocomposites with metal oxide nanoparticles of SiO_2_ and Al_2_O_3_. An efficiency of 80% of methylene blue at the equilibrium was reported at 60 min [31].

Figure 7b shows the adsorption of methyl orange (MO) in relation to time. Neat Nylon 6 fabric shows an adsorption efficiency of 22%. Nylon 6/C20A 1.5% shows a rapid adsorption in the first 10 min, reaching 60% of MO adsorption efficiency. In addition, at the equilibrium, 78% adsorption efficiency is reached. Nylon 6/C20A 2% nanocomposite reaches an adsorption efficiency of 92% at the equilibrium. This value is higher due to larger vacant sites being occupied by adsorbate molecules [32] because of the higher content of nanoclay. Additionally, Figure 7a,b show images from before and after dye adsorption of the fabric.

Table 5 and Table 6 show the linear models of the Langmuir and Freundlich isotherms which determine the adsorption capacity and the type of adsorption. Both tables show that the R^2^ of the Langmuir isotherm is higher than the Freundlich isotherm. Therefore, the adsorption process of Nylon 6/C20A non-woven fabric is better described by the monolayer isotherm. These results agree with Andrade et al., who studied the adsorption of “uremic toxins or MB and MO” on PLA/C20A nanocomposite. The data were better adjusted to the Langmuir model, indicating that the adsorption occurred in a single layer [20]. As shown in Table 5, the samples Nylon 6/C20A 0.25% and Nylon 6/C20A 0.5% show heterogeneous behavior; having a low concentration of the nanoclay, the dispersion allows multilayer adsorption.

Table 6 shows the results of the adsorption isotherms. All the samples with different concentrations of nanoclay conform to the Langmuir isotherm, a monolayer is formed with the dye molecules on the surface of the non-woven fabric, as well as the MB. It is a cyclic structure that can form electrostatic interactions with the amino groups of the modified nanoclay. The Nylon 6 sample without nanoclay fits better with the Freundlich isotherm, forming multilayers of the MB on the surface of the polymer matrix.

### 3.6. Reusability of Non-Woven Fabric Nylon 6/C20A for the Adsorption of Dyes and Uremic Toxins

**Figure 8:**
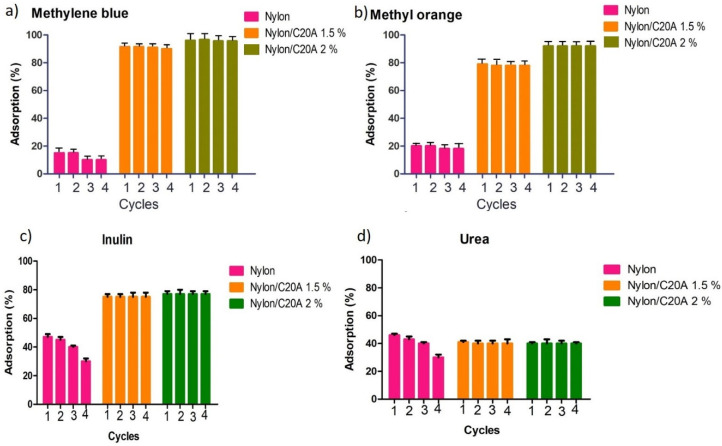
Reusability test for (**a**) MB, (**b**) MO, (**c**) inulin, and (**d**) urea adsorption.

After the first process of adsorption, non-woven fabrics were recovered and dried at room temperature. This process does not require a regeneration with any solvent or acid solution, which is an advantage of the elaborated material, because most nanoparticle composites need a regeneration process. Figure 8a,b show the methylene blue and methyl orange adsorption capacity of Nylon 6/C20A non-woven fabric during four cycles. Even after four adsorption cycles, Nylon 6/C20A 1.5% and Nylon 6/C20A 2% maintain an adsorption efficiency above 90%. Figure 8c shows the adsorption efficiency after four cycles. In the case of neat Nylon, a decrease in adsorption is observed from cycle 3. However, the adsorption efficiency remains constant for Nylon 6/C20A 1.5% and Nylon 6/C20A 2% at 75 and 77%, respectively. Figure 8 shows the urea adsorption. The adsorption efficiency for neat Nylon is 47%, decreasing after the third cycle. By contrast, for Nylon 6/C20A 1.5% and Nylon 6/C20A 2%, the adsorption efficiencies do not decrease and remain constant after four cycles.

The adsorption efficiency % of non-woven Nylon 6/C20A for uremic toxins and dyes was compared with other adsorbents based on nanocomposite reported in the literature (see Table 7). In the last study with carbon-based nanoparticles, the maximum adsorption efficiency was 80 to 95% for uremic toxins. This is because the surface area is greater and due to the chemical modification of the nanoparticles [11]. Andrade et al. reported an adsorption efficiency for urea of 65% and 97% for methylene blue with the same modified nanoclays used in this study. It is observed that compared to this study, the material is more efficient with the PLA polymer, which is due to the polymer structure that has carboxylic groups that allow efficient adsorption [20]. Le et al. studied the polyamide functionalized with Fe and observed an adsorption efficiency for urea of 85% [33]. Non-woven based on Nylon 6/ZnO and polyester-supported cuprous oxide/reduced graphene oxide both report above 90% for the adsorption of methylene blue [17,19]. Also, composites such as chitosan/polyvinyl alcohol/zeolite and polyethylene oxide/bentonite/polyaniline present values above 90% for MO adsorption, like this study [34,35].

### 3.7. Antibacterial Activity

Gram-negative bacteria such as *E. coli* are widely used to assess antibacterial activity and ecological safety in materials because their presence in environmental samples, food, or water indicates recent fecal contamination or poor sanitation practices. In addition to the materials obtained in this study, an antibiotic like gentamicin was used as a control.

**Figure 9:**
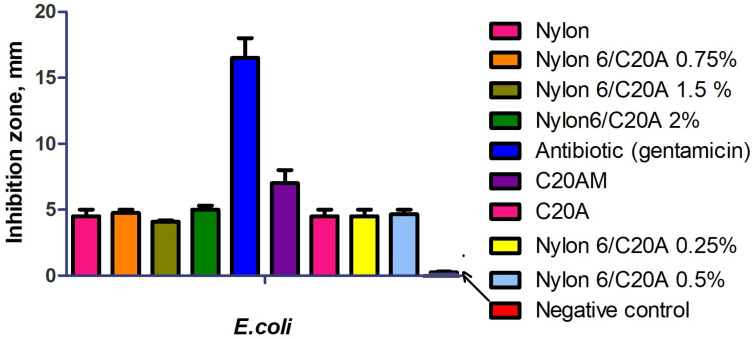
Effect of Nylon 6/C20A non-woven antibacterial activity against *E. coli*.

Figure 9 shows the antibacterial activity of non-woven Nylon 6/C20A against *E. coli*. The antibiotic presents an inhibition zone of 13.5 mm; conversely, the non-woven Nylon 6/C20A fabric at different contents shows an inhibition zone of 4 ± 0.5, presenting slight antibacterial activity against *E. coli*. These results agree with those of Latwinska et al., who reported that PP and PLA non-woven fabrics have no antimicrobial activity against *E. coli* and *S. aureus*. However, PP/PLA/CuO.SiO_2_ composites presented antibacterial activity [36].

The antibacterial activity of neat Nylon 6 non-woven fabrics was presented in previous work [19]. The modified fabrics have a greater inhibition zone than the pristine nanoclay. This observation agrees with the study of Nigmatullin et al., where nanoclays Cloisite 20A, 15A, and 30A were modified with biocides and cationic surfactants. Although they attribute the antibacterial efficiency to modifying agents, the inhibition zones presented by these nanoclays were small [37].

**Figure 10:**
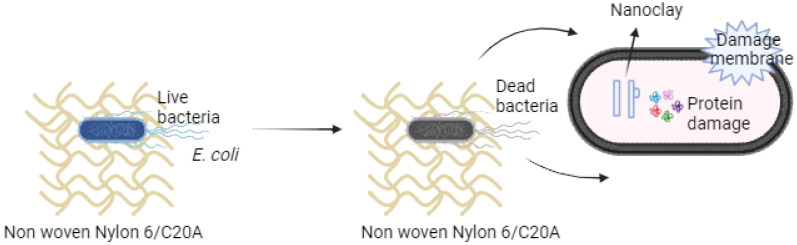
Diagram of the possible antibacterial mechanism of non-woven fabric.

The possible antibacterial mechanism of nanoclays may be due to damage to the cell wall and membrane and the bacteria proteins, as shown in Figure 10. The bacterial cell membrane has a negative surface charge and the nanoclays have a positive charge due to the amino groups with which it was chemically modified. Therefore, they bind to the membrane of the bacteria through electrostatic interactions, then cause damage to the membrane and interact with biomolecules of the bacteria such as proteins that cause the death of the bacteria [38].

## 4. Conclusions

Based on the results previously reported, this study produced non-woven fabrics based on polymer nanocomposites of Nylon 6 and modified nanoclay with amino groups. The XRD results showed a displacement of 2Ɵ angles due to the phase shift of Nylon 6. The SEM images showed that the mean diameter of the fiber decreased as the nanoclay content increased. The mechanical tests showed that the sample with 0.5% of modified nanoclay augmented the fiber tear strength to 2.135 N. The inulin adsorption process occurred mainly after 15 min, presenting efficiencies of 75 and 74%. The adsorption capacity for methylene blue and methyl orange was 90%, even after four cycles, and the modified nanoclay non-woven fabrics showed antibacterial activity against *E. coli*.

As a future perspective, when developing this research on non-woven fabric with Nylon 6 and modified nanoclay, the objective is to obtain the necessary knowledge, to have a membrane that can have adsorption properties of uremic and antibacterial toxins, and then test it in environments that help us see their feasibility and selectivity for use in the medical field.

## 5. Patents

MX/a/2022/013972.

## Figures and Tables

**Table 1:** Fiber diameter of non-woven fabric at different concentrations.

Sample	Fiber Diameter (µm)
Nylon 6	16 and 17 ± 1.5
Nylon 6/C20A 0.25%	16 ± 0.7
Nylon 6/C20A 0.5%	15 ±1.8
Nylon 6/C20A 0.75%	13 ± 2.1
Nylon 6/C20A 1.5%	12 ± 2.4
Nylon 6/C20A 2.0%	12.7 ± 1.76

**Table 2:** Mechanical properties of non-woven fabrics.

Sample	Tear Force (N)	Maximum Breaking Strength (N)
Nylon 6	1.734	13.24
Nylon 6/C20A 0.25%	1.334	1.19
Nylon 6/C20A 0.5%	2.334	4.73
Nylon 6/C20A 0.75%	1.4243	5.56
Nylon 6/C20A 1.5%	1.7348	3.66
Nylon 6/C20A 2.0%	1.7792	3.08

**Table 3:** Isotherm constants and correlation coefficients for urea adsorption on Nylon 6/C20A non-woven fabric at different concentrations.

Sample	Langmuir			Freundlich		
	k	q_max_	R^2^	n	K_F_	R^2^
Nylon	0.02	3.67	0.9948	0.35	12.46	0.9994
Nylon 6/C20A 0.25%	0.04	8.11	0.9986	0.30	7.09	0.9989
Nylon 6/C20A 0.5%	0.4	8.10	0.9906	0.31	7.00	0.9989
Nylon 6/C20A 0.75%	0.11	1.44	0.9996	0.69	9.10	0.9997
Nylon 6/C20A 1.5%	0.01	1.60	0.9903	0.65	8.96	0.9995
Nylon 6/C20A 2%	0.01	1.47	0.9980	0.68	9.12	0.999

**Table 4:** Isotherm constants and correlation coefficients for inulin adsorption onto Nylon 6/C20A non-woven fabric at different concentrations.

Sample	Langmuir			Freundlich		
	k	q_max_	R^2^	n	K_F_	R^2^
Nylon	0.16	25.8	0.999	1.12	11.4	0.9996
Nylon 6/C20A 0.25%	0.06	0.94	0.9996	2.71	18.52	0.9994
Nylon 6/C20A 0.5%	0.006	0.7	0.9999	3.08	20.52	0.9992
Nylon 6/C20A 0.75%	0.04	1.13	0.9998	3.07	20.46	0.9994
Nylon 6/C20A 1.5%	0.059	66	0.9999	3.23	21.30	0.9994
Nylon 6/C20A 2%	0.042	1.24	0.9999	3.05	20.35	0.9995

**Table 5:** Adsorption isotherm parameters for MO dye adsorbed onto Nylon 6/C20A.

Sample	Langmuir			Freundlich		
	k	q_max_	R^2^	n	K_F_	R^2^
Nylon	9.3	312	0.9976	4.8	30.4	0.8799
Nylon 6/C20A 0.25%	8.3	113	0.9003	0.18	50.5	0.934
Nylon 6/C20A 0.5%	1.5	132	0.9317	4.9	52	0.9574
Nylon 6/C20A 0.75%	2.2	210	0.9886	4.0	51	0.9297
Nylon 6/C20A 1.5%	0.29	432	0.9994	2.6	8.11	0.9977
Nylon 6/C20A 2%	2	67.6	0.9998	6.5	14.77	0.9945

**Table 6:** Adsorption isotherm parameters for MB dye adsorbed onto Nylon 6/C20A.

Sample	Langmuir			Freundlich		
	k	q_max_	R^2^	n	K_F_	R^2^
**Nylon**	1.20	196.92	0.9387	9.61	52.17	0.9817
**Nylon 6/C20A 0.25%**	0.03	199	0.9990	0.52	2.77	0.9905
**Nylon 6/C20A 0.5%**	1.5	186	0.9953	1.25	9.47	0.9545
**Nylon 6/C20A 0.75%**	1.66	193	0.9973	1.4	6.09	0.9038
**Nylon 6/C20A 1.5%**	0.23	151.4	0.9933	4.6	11.32	0.9448
**Nylon 6/C20A 2%**	0.19	343	0.9985	9.7	20.11	0.9565

**Table 7:** Comparison of the adsorption efficiency % for uremic toxins and dyes.

Material	Uremic Toxins (Adsorption %)		Dyes (Adsorption %)		References
	Urea	Inulin	Methylene Blue	Methyl Orange	
Nylon 6/CB	80–90	80–85	----	----	[11]
PLA/C20A nanoclay	65	----	97	----	[20]
Nylon 6/ZnO	----	----	93	----	[19]
Polyamide functionalized with Fe-based metal–organic	85	----	----	----	[33]
Non-woven polyester fabric-supported cuprous oxide/reduced graphene oxide	----	----	96	----	[17]
Chitosan/polyvinyl alcohol/zeolite electrospun composite	----	----	----	95	[34]
Polyethylene oxide/bentonite/polyaniline	----	----	96	94	[35]
Nylon 6/C20A 1.5%	40	75	90	78	This study
Nylon 6/C20A 2%	40	74	90	92	This study

## Supplementary Materials

The following supporting information can be downloaded at: https://www.mdpi.com/article/10.3390/ma17123007/s1, Figure S1: SEM of nonwoven fabric at 100x of nylon and nylon6/C20A a different concentrations.
