# Supplementary material for: Functional Technical Textile-Based Polymer Nanocomposites with Adsorbent Properties of Toxins and Dyes also Have Antibacterial Behavior
Source: Materials (Basel). 2024 Jun 19;17(12):3007. doi: 10.3390/ma17123007 (PMC11205333; doi:10.3390/ma17123007)

Scanning electron microscopy (SEM)

SEM of nonwoven fabric at 100X of A) Nylon 6, B) Nylon 6/C20A 0.25%, C) Nylon 6/C20A 0.50%, D) Nylon 6/C20A 0.75%, E) Nylon 6/C20A 1.50%, F) Nylon 6/C20A 2.00%

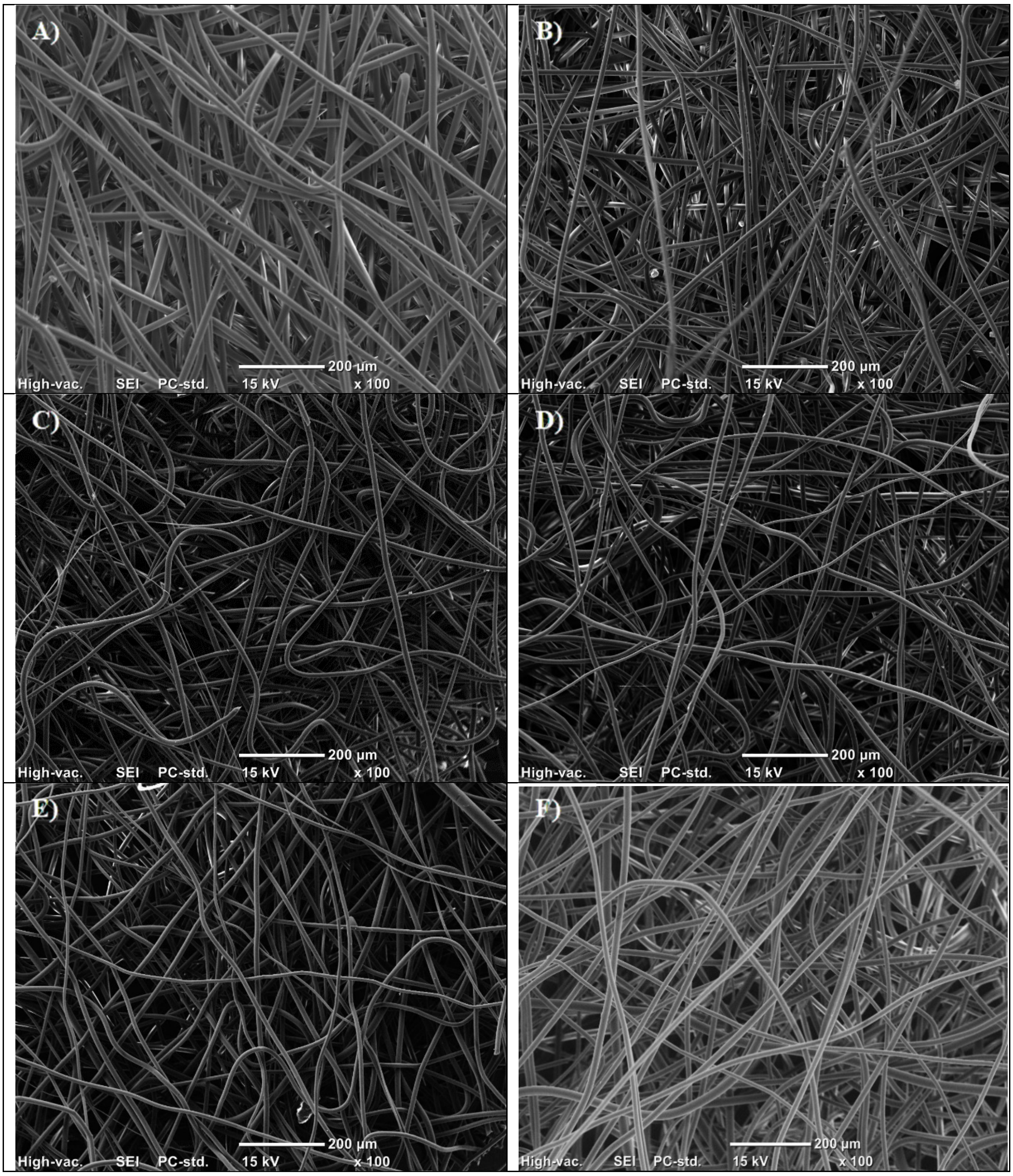

Supplement: Supplementary file 1 [file materials-17-03007-s001.zip › materials-3026148-supplementary.pdf]
